# Mind the influence of arterial oxygen tension on central venous oxygen saturation

**DOI:** 10.1186/s13054-014-0569-y

**Published:** 2014-10-16

**Authors:** Huai-wu He, Da-wei Liu, Yun Long, Xiao-ting Wang

**Affiliations:** Department of Critical Care Medicine, Peking Union Medical College Hospital, Chinese Academy of Medical Sciences, 1 shuaifuyuan, Dongcheng District, Beijing, 100730 China

We read with great interest the study by Hernandez and colleagues published in *Critical Care* [1]. The study showed that central venous oxygen saturation (ScvO_2_) increased significantly in response to emergency intubation, and the authors suggested that the early normalization of ScvO_2_ after intubation might not be reliable to reflect successful resuscitation. However, they might have ignored the influence of arterial oxygen tension (PaO_2_) on ScvO_2_.

It is well known that when arterial oxygen saturation is approaching 100%, the increase in oxygen delivery would be limited in response to the increase of PaO_2_. However, a very high PaO_2_ could significantly influence ScvO_2_ even if arterial oxygen saturation reaches 100%. Pre-oxygenation may result in a very high PaO_2_ in the emergency intubation, so PaO_2_ should be taken as a potentially confounding factor. Very high PaO_2_ (about 288 mmHg) has a more significant and consistent effect on ScvO_2_ than a relevant change in cardiac index (>10%) [2]. Moreover, a decrease in the whole body oxygen consumption under hyperoxia has been reported in critically ill patients [3], and animal studies also noted that hyperoxia could result in the redistribution of cardiac output [4]. Recently, Legrand and colleagues documented that an increase in PaO_2_ could increase ScvO_2_ without increasing oxygen delivery [5].

It is worth paying attention to the impact of PaO_2_ on ScvO_2_ in the management of critically ill patients.

## Abbreviations

PaO_2_: Arterial oxygen tension
ScvO_2_: Central venous oxygen saturation
